# Outcome measures in psoriatic arthritis: Where next?

**DOI:** 10.1002/msc.1692

**Published:** 2022-11-10

**Authors:** Simon Hackett, Laura C. Coates

**Affiliations:** ^1^ Nuffield Department of Orthopaedics, Rheumatology and Musculoskeletal Sciences University of Oxford Botnar Research Centre Oxford UK

**Keywords:** outcomes measurements, PROM, psoriatic arthritis

## Abstract

**Objective:**

To provide an overview of commonly used outcome measure in psoriatic arthritis (PsA).

**Background:**

PsA is a heterogenous inflammatory arthritis, associated with psoriasis that affects between 0.1% and 2% of the population and approximately one in three patients with psoriasis. Psoriatic arthritis places a significant burden on patients' overall quality of life and is associated with a range of comorbidities. Although assessment of patients and monitoring of symptoms has greatly improved over the last 2 decades, capturing disease activity in this multisystem disease remains challenging. Previous efforts have traditionally focussed on assessment of individual disease domains, however recent evidence suggests that composite measurements, particularly those incorporating patient reported outcomes may not only help monitor disease activity more accurately, but also help in accurately validating therapy outcomes in PsA patients.

**Purpose:**

This review discusses currently used outcome measurements in PsA and also highlights the importance of emerging measurements such as biomarkers and their possible role in capturing treatment response.

## 1 INTRODUCTION

Psoriatic arthritis (PsA) is a heterogenous condition with musculoskeletal involvement, manifesting as a variety of symptoms including arthritis, dactylitis, enthesitis and axial involvement (Coates and Helliwell, 2017). In addition to musculoskeletal symptoms, patients with PsA have other symptoms such as nail disease, which affects between 63% and 83% of patients (Nieradko‐Iwanicka, 2017). Along with the varied nature of symptoms, the repercussions on patients are equally as diverse. Patients with PsA have an increased risk of comorbidities such as cardiovascular disease, metabolic syndromes, depression, inflammatory bowel disease and anxiety. In order to measure the impact of PsA on patients, a range of outcomes measures have been developed to ascertain disease impact and help monitor response to treatment in both clinical trials and the real‐world settings.

Despite the development of a range of outcome measures for patients, agreement as to the exact measure that should be utilized remains a challenge. Recent recommendations from the TIght Control Of Psoriatic Arthritis (TICOPA) trial have highlighted the need and importance of regular assessment of disease activity by clinicians, as well as the critical role patients play in capturing their own concerns (Coates et al., 2015). To capture these outcome measurements, The Group for Research and Assessment of Psoriasis and Psoriatic Arthritis (GRAPPA)‐Outcome Measures in Rheumatoid Arthritis Clinical Trials (OMERACT) PsA working group developed a Core Domain Set in 2007 which was updated in 2014 in light of emerging understanding of the pathophysiology of PsA.

These outcome measures not only help guide clinicians in monitoring disease activity in patients, but also help objectively gauge responses to treatment and provide an on‐going source of real‐world evidence.

This review will examine the current outcomes that have been developed for PsA and their clinical utility, as well as providing an overview of emerging tools for assessing outcomes in patients, including the use of biomarkers.

## 2 CURRENT OUTCOMES MEASUREMENTS

Current outcome measures primarily focus on assessing either individual disease domains or composite measurements, which aim to combine multiple outcome assessments into one score. There are 6 principal domains that have previously been identified by GRAPPA in 2015 to help guide treatment and management of patients: arthritis, axial disease, enthesitis, dactylitis, and psoriatic skin and nail disease (Coates et al., 2016).

### 2.1 Arthritis

Joint inflammation is a key feature of PsA. Although a common feature, joint inflammation is often heterogenous, especially in comparison to rheumatoid arthritis (RA), with a wider variety of joints involved. Given the importance of joint health on quality of life, assessing and monitoring arthritis forms an important component of rheumatological assessment of PsA patients.

The 28‐joint Disease Activity Score (DAS28), also used for the assessment of RA, has been used for assessment of arthritis in PsA patients (Kalyoncu et al., 2016). However, in order to capture more joints, the Disease Activity index for PSoriatic Arthritis (DAPSA) was developed using the 66/68 joint count instead of the 28 joint count. Along with joint count, DAPSA also takes into account patient pain, C reactive protein (CRP) level and pain score. Although superior to using the DAS28 in isolation, it is not perfect in capturing outcomes over the disease course, with a relatively recent study showing that both responsiveness and longitudinal validity of most outcomes were inferior for DAS28 and DAPSA (Wervers et al., 2019).

American College of Rheumatology 20% (ACR20) response incorporates a 68/66 joint count, alongside assessment of the following five criteria: patient global assessment, physician global assessment, functional ability measure [most often Health Assessment Questionnaire (HAQ)], visual analogue pain scale, and erythrocyte sedimentation rate (ESR) or CRP. A 20% improvement in the number of tender and number of swollen joints, and a 20% improvement in 3 of the 5 aforementioned criteria constitute an ACR20 response (Felson & LaValley, 2014). Although used as an outcome measure in clinical trials, performing ACR20 in the clinic is not feasible due to the time and challenges inherent in this.

### 2.2 Axial disease

Pure axial involvement is seen in about 5% of cases of PsA but axial involvement (defined by symptoms and/or radiography), along with peripheral involvement, may be seen in over 50% of cases (Helliwell, 2020). Despite over half of patients being affected by axial disease, diagnosis, measuring and monitoring is significantly challenging.

Measurement of axial disease has traditionally ‘piggybacked’ outcome measures used for ankylosing spondylitis (AS), such as the Bath AS Disease Activity Index (BASDAI). The BASDAI consists of a 0–10 scale measuring pain, fatigue and discomfort in response to 6 questions including: fatigue, spinal pain, arthralgia/welling, enthesitis, morning stiffness duration and morning stiffness duration. Although the BASDAI is routinely used to measure disease in axial PsA, it correlates equally well with the patient global scores in patients who have both axial and pure peripheral PsA, thus the BASDAI should be used with caution for evaluating disease activity in axial PsA (Fernández‐Sueiro et al., 2010).

Five out of the six questions in the BASDAI are not specific to axial disease. A recent study evaluated the specificity of BASDAI for axial disease in PsA compared to PsA without axial disease by examining differences in baseline scores, change in scores after therapy initiation, and responsiveness between the two groups (Reddy et al., 2020). The authors noted that the BASDAI functions well as a broad measure of disease activity and symptoms in PsA patients but may not be specific to axial involvement.

The Ankylosing Spondylitis Disease Activity Score (ASDAS) is a composite disease activity score for AS which uses a number of BASDAI questions and incorporates either C‐reactive protein (CRP) or ESR values. Both have been evaluated in PsA and a cross‐sectional study showed that the ASDAS might also be a valuable tool to measure disease activity and to define clinical remission in PsA (Hermann et al., 2016) although specificity to spinal disease remains an issue. Both BASDAI and ASDAS are used routinely in axial PsA given a lack of alternatives, but for specific evidence of axial involvement, imaging is required.

### 2.3 Enthesitis

Enthesitis is a common clinical feature of PsA, which is characterized by inflammation at the site of insertion of tendons, ligaments and joint capsule fibres into bone (Mease, 2020).

The Leeds Enthesitis Index (LEI) scores three sites bilaterally: the lateral elbow epicondyle, medial femoral condyle and Achilles tendon insertion. Clinicians assess the presence or absence of pain/tenderness by application of 4 kg of pressure to the site. A positive result at a site is given a score of 1, with a total score of 6 achievable. Although there are limited data on validity, the reliability of the test is good, with a correlation among clinical enthesitis indices (Ogdie et al., 2020). The LEI has been used as an outcome measure in several clinical trials, trials including the SPIRIT‐P1 and ‐P2 (Gladman et al., 2019).

The Spondyloarthritis Consortium of Canada (SPARCC) enthesitis score, another tool to assess enthesitis, was created as a measure for enthesitis in spondyloarthritis in general and is not limited to assessing enthesitis in PsA or AS. Enthesitis is scored bilaterally across eight sites, following application of 4 kg of pressure, including: medial epicondyle, lateral epicondyle, supraspinatus insertion into greater tuberosity of humerus, greater trochanter, quadriceps insertion into superior border of patella, patellar ligament insertion into inferior pole of patella or tibial tubercle, Achilles tendon insertion into calcaneum and plantar fascia insertion into calcaneum (Maksymowych et al., 2009). As with the LEI, although there are limited data on validity, the reliability of the test is good, with a correlation among clinical enthesitis indices (Ogdie et al., 2020). The SPARCC has also been used as a clinical trial outcome, including assessing the clinical utility of clazakizumab and ustekinumab in PsA (Araujo et al., 2019; Mease et al., 2017).

### 2.4 Dactylitis

Dactylitis, a hallmark feature of PsA, occurs in up to nearly 50% of patients, and is associated with more erosive and aggressive disease phenotypes (Kaeley et al., 2018). Dactylitis often occurs asymmetrically and generally involves the feet more commonly than the hands.

Dactylitis is routinely assessed in clinical trials with most studies using a simple count of dactylitic digits. The Leeds Dactyilitis Instrument (LDI) provides a more detailed assessment of dacylitis. The LDI measures the ratio of the circumference of the affected digit to the circumference of the digit on the opposite hand or foot, with a minimum difference of 10% used to define the presence of dactylitis (Healy & Helliwell, 2008). The score takes less than 10 min to complete, has no floor or ceiling effects and correlates strongly with outer measures of dactylitis (Ogdie et al., 2020). The LDI has been used as an outcome measure in a range of trials including the SPIRIT‐P1 and ‐P2 (Gladman et al., 2019).

### 2.5 Skin

Skin disease, namely psoriasis, has a significant impact on quality of life of PsA patients (Kasiem et al., 2022). To date, a significant range of outcome measures have been proposed to assess skin disease activity in PsA patients. In keeping with the pattern of disease, commonly used psoriasis scoring systems classically used in dermatology practice, such as Psoriasis Area Severity Index (PASI) have been used to assess patients and is routinely used in clinical trials to help determine treatment efficacy within this disease domain (Edwards et al., 2016; van der heijde et al., 2018). The PASI score is determined through assessment of psoriasis lesions and scores intensity of erythema, induration and desquamation a score of between 0 and 4, taking into account the percentage of the body that is affected (Feldman & Krueger, 2005). Although the PASI is relatively easy for clinicians to use, it is not usually calculated in patients with <3% body surface area (BSA) involvement. As a result, measuring a PASI score in some patients, including those in clinical trials, may be difficult and may underrepresent skin disease in some patients (Mease, 2011). Although the PASI proves a useful tool in rheumatological practice, the amount of time to calculate the score is also on occasion a barrier to use.

Alongside PASI, other skin assessment tools are frequently used to assess psoriasis in PsA patients. The Physicians Global Assessment (PGA) is an average assessment of all psoriatic lesions, based on erythema, scale and induration. It neither quantifies BSA nor evaluates individual lesion locations (Bożek & Reich, 2017). The static variation of the PGA measures disease at a single time point, with psoriasis lesion scored between 0 = clear 1 = almost clear, 2 = mild, 3 = moderate, 4 = severe, 5 = very severe. Although the score is quick to perform and calculate, the reliability of PGA among rheumatologists is lower than that of the PASI, suggesting scores may not always truly reflect disease activity in PsA patients (Chandran et al., 2009).

The BSA score has traditionally been used to determine the total approximate area of the body affected by psoriasis. The BSA scoring system has also been combined with the PGA into the PGAxBSA. Studies have shown strong correlation with PSA scores, along with good responsiveness (Walsh et al., 2018). PGAxBSA scores have been used to determined treatment response in the real‐world setting, including response to IL‐17 therapy in psoriasis patients (Armstrong et al., 2021).

### 2.6 Nail

Nail involvement is common in PsA patients, with rates of up to 80% reported in the literature (Crowley et al., 2015; Reich, 2009). There exists a significant correlation between PsA and nail changes: the presence of the latter associated of the development of arthritis. The assessment of nail disease is therefore of significant clinical importance.

The Nail Psoriasis Severity Index (NAPSI) is an assessment tool that is used primarily as an outcome measure in clinical trials. Each nail is given an eight‐point score, according to the features present within the nail and nail bed. Each fingernail is scored, yielding a maximal score of 80 or up to 160 if the toenails are also included (Rich & Scher, 2003). Nail Psoriasis Severity Index was used as an outcome measure in the SPIRIT‐P1 trial (Mease et al., 2016), owing to its good reliability amongst trained clinicians (Aktan et al., 2007).

Other nail measurement commonly used include the modified NAPSI (mNAPSI) (Cassell et al., 2007) which is considered simpler and more reliable than the original NAPSI. Scoring takes less than 5 min with studies showing excellent reliability, whilst maintain the validity and clinical relevance of NAPSI (Mease, 2011).

**FIGURE 1:**
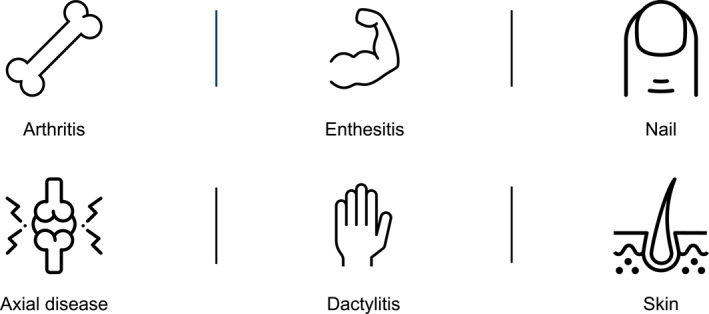
Domains often examined as outcome measures in psoriatic arthritis

A view of all domains commonly assessed is shown in Figure 1.

### 2.7 Composite outcome measurements

Composite outcome measurements aim to overcome the main hurdle of single domain measurements by capturing outcomes across multiple domains in order to measure holistic disease burden.

The minimal disease activity (MDA) criteria were developed in 2010 through analysis of patient profiles in an observational PsA patient database (Coates et al., 2010). Sixty experts classified whether these patients met the standard of MDA, with summary statistics created for possible cut‐off points for defining MDA. Seven items were used to form the basis of MDA, with patients meeting 5 or more of the 7 criteria being deemed as achieving MDA. These criteria include: tender joint count ≤1, swollen joint count ≤1, enthesitis count ≤1, PASI ≤1 or BSA ≤3, patient global visual analogue score (VAS) ≤20mm, patient pain VAS ≤15mm and health assessment questionnaire (HAQ) ≤0.5. The MDA criteria have been shown to have prognostic value in terms of quality of life and radiographic, as well as corresponding highly with a level of symptoms that is acceptable to patients (Coates et al., 2018; Querio et al., 2017). With respect to responsiveness, MDA criteria have shown good responsiveness to change and are able to differentiate between treatment and placebo (Ogdie et al., 2020). The MDA criteria have also helped guide the treat‐to‐target approach, as part of the TICOPA trial (Coates et al., 2015).

In 2009, a GRAPPA working group aimed to develop comprehensive recommendations for the treatment of the various clinical manifestations of PsA based on evidence obtained from a systematic review of the literature and from consensus opinion amongst dermatologists and rheumatologists (Ritchlin et al., 2009). This work formed the basis of the composite measure Composite Psoriatic Disease Activity Index (CPDAI) (Mumtaz et al., 2011). CPDAI assesses each PsA domain in the context of both disease activity and also impact of disease on patient function and QoL. The measurements are summated to generate a score between 0‐15. The CPDAI has been well validated and is relatively easy to use in trials or clinical practice but takes a little longer to assess than other composites with more questionnaires required (Ogdie et al., 2020). CPDAI has shown good responsiveness to change and differentiates between treatment and placebo, and also shows good responsiveness in LOS. The effect size has been shown to be smaller than PASDAS in clinical trial post‐hoc analysis (Ogdie et al., 2020).

In 2013, a GRAPPA working group also developed 2 other composite indices through GRAPPA Composite Exercise (GRACE). This longitudinal study consisting of 503 PsA patients collected a range of data at 0, 3, 6 and 12 months. Any treatment changes were noted at each visit, with treatment escalation representing as a surrogate for increased disease activity.

The PsA Disease Activity Score (PASDAS) was developed by multiple linear regression analysis of the GRACE cohort to determine the weighted index of each constituent domain. The PASDAS includes both clinician‐reported (such as domain‐specific symptoms and CRP level) and patient‐reported outcome measures combined into a composite index. The GRACE index contains eight domain measures transformed using desirability functions and then combined (Helliwell et al., 2013). Both the PASDAS and GRACE index have shown to correlate with patient reported outcome measurements, as well as radiographic disease progression and post‐hoc analysis of previously undertaken randomised controlled trials with secukinumab (Coates et al., 2018; Helliwell & Kavanaugh, 2014). With respect to performance, PASDAS has shown good responsiveness to change in clinical trials and has ability to differentiate between treatment and placebo. The ability of the PASDAS to demonstrate good responsiveness and discriminative ability was shown in the RAPID‐PsA trial (Helliwell et al., 2014).

Other newer composite measurements, such as the PsA 5‐thermometer scales (PsA‐5 Ts) have also been developed in order to assess disease activity (Salaffi et al., 2022). The PsA‐5T is a simple instrument with five “thermometers” that incorporate pain, fatigue, physical function, skin disorders, and depression into a single assessment of disease activity. Upon analysis in a small group of PsA patients, the PsA‐5 Ts had same discriminant validity as the DAPSA, CPDAI and PASDAS, suggesting it may be potentially as useful as these measures, with the advantage of being potentially quicker and easier to administer. The PASDAS has, however, yet to undergo further significant validation in extensive clinical trials or analyses.

Composite measurements offer significant benefits to single domain measurements and offer the unique ability to capture disease activity across multiple domains, from both a clinician and patient perspective. Their slightly cumbersome and time‐consuming nature however does potentially make their routine use in the clinic a challenge.

## 3 PATIENT REPORTED OUTCOME MEASUREMENTS

In recent years, there has been a shift towards incorporating PROMs to assess disease activity from a patient‐centric perspective. Patient reported outcome measurements are standardized questionnaires that collect information on health outcomes directly from patients, including symptomology, health‐related quality of life, functional status and other disease‐specific measurements (Churruca et al., 2021). They can be broadly split into two categories: generic and disease specific. Generic measurements capture outcomes across a broad range of diseases and allow for comparison, whilst disease‐specific PROMs facilitate capture of disease‐specific parameters.

In addition to their use at the patient level to monitor the progress of individual patients, PROM data have the potential to help assess a range of factors that previous scoring systems are unable to capture by incorporating more of the impact of disease from the patient perspective. Patient reported outcome measurement tools have been validated in a wide range of diseases including stroke (Reeves et al., 2018), chronic obstructive pulmonary disease (Jahagirdar et al., 2013), RA (Hendrikx et al., 2016) and PsA (Chakravarty et al., 2021; Gossec et al., 2014).

Historically, PROM measurements in PsA were ‘borrowed’ from other diseases, particularly other rheumatological conditions such as RA or AS (Orbai & Ogdie, 2016). Patient reported outcome measurements are often assessed across disease activity domains, such as pain, skin and HRQoL. Some of these outcomes can be assessed with generic, non‐PsA specific measurements such as the visual analogue scale (VAS) for pain or the Medical Outcomes Study Short Form‐36 (SF‐36) for HRQoL. However, these tools often fail to capture specific disease‐specific factors such as disease variation and something else. Importantly, certain PROMs for other rheumatological conditions, such as the Arthritis Impact Measurement Scale have failed validation for PsA (Duffy et al., 1992), with others such as the SF‐36 showing only moderate correlation with clinical indicators of function, pain and arthritis and poor correlation with disease severity (Husted et al., 1997). One of the reasons that these tools fail may be due to PsA having both rheumatic and dermatological impacts on patients.

There has, therefore, been much effort to develop PsA‐specific PROMs. Several have been developed and subsequently validated. They are discussed below.

### 3.1 PsA quality of life (PsAQoL)

The PsAQoL is a PsA‐specific PROM which measures the QoL in patients and has since been extensively validated since its initial inception (McKenna et al., 2004). The questionnaire was formulated from qualitative interview conducted with patients with PsA. Analysis of the interviews identified a 51‐item questionnaire which was subsequently reduced to 35 items. Ultimately, a 20‐item version of the PsAQoL was shown to have excellent test‐restest reliability and validity. A further study in 2008 in 28 patients following change in disease modifying therapy was used to examine the sensitivity and response to change of the PsAQoL (Healy & Helliwell, 2008). Findings from this study demonstrated strong correlation with other PROM such as HAQ and Patient Global.

The PsAQoL has shown utility in both clinical practice and trial settings, including the DISCOVER‐1 trial, examining the efficacy and safety of guselkumab in PsA patients (Ritchlin et al., 2021).

The PsAQoL has since been translated into other languages and validated in a range of populations including Sweden, Brazil and Holland, demonstrating its usefulness in broad populations (Billing et al., 2010; Gonçalves et al., 2021; Wink et al., 2013).

### 3.2 PsA impact of disease (PsAID) questionnaire

**FIGURE 2:**
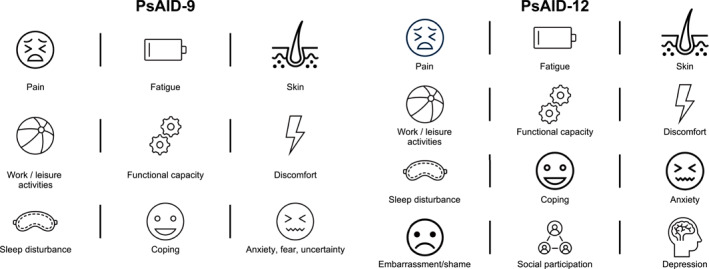
Domains examined in PsAID‐9 and PsAID‐12

Perhaps the most well‐known PROM tool for PsA clinicians is the PsA Impact of Disease (PsAID) questionnaire (Gossec et al., 2014). The PsAID was developed by a EULAR taskforce in 2014 consisting of both patients and clinicians from 13 countries with the aim of providing a tool to assess the impact of PsA from a patient perspective (Coates et al., 2020). Two questionnaires were ultimately developed: one for use in day‐to‐day clinical practice (PsAID‐12) and one for use in clinical trials (PsAID‐9), domains of which are shown in Figure 2. Both questionnaires, which are free to use, capture data across domains of health: 12 in the PsAID‐12 and 9 in the PsAID‐9. Domains were chosen following a literature review summarizing published criteria, measures and questionnaires using in PsA trials (Gossec et al., 2014; Palominos et al., 2012), with participants identifying key domains of health in terms of quality of life, based on their own experiences. The domains of health assessed in the PsAID‐12 are grouped into 3 categories of impact: physical impact; impact related to skin; and psychological and social impact (Tälli et al., 2016).

The questionnaire was initially validated through an international cross‐sectional, longitudinal study in 474 patients with a confirmed diagnosis of PsA by a rheumatologist (Gossec et al., 2014). The initial validation showed that both the PsAID‐9 and PsAID‐12 were highly reliable with significant correlation with patient global assessment.

A further validation study was undertaken in 2018 to examine the utility of PsAID as a single‐item outcome measure in clinical practice, with a focus on estimating the minimally important difference for improvement and exploring the potential of individual components (Holland et al., 2018). Across 129 patients with PsA in the UK, the authors examined both PsAID‐9 and PsAID‐12 against established PROMs including Health Assessment Questionnaire‐Disability Index (HAQ‐DI), EuroQol‐5D (EQ‐5D‐5 L) Questionnaire, Psoriatic Arthritis Quality of Life (PsAQoL) Questionnaire, Dermatology Life Quality Index, amongst others. The authors concluded that the PsAID is a reliable, feasible and discriminative measure in patients with PsA, with the ability to measure both stable and active disease in PsA patients.

The authors recognized that additional data from interventional clinical trials, such as patients undergoing treatment with biologics, will provide further validation and explore its utility in detecting meaningful changes following treatment.

The PsAID has since been translated into other languages, such as Portuguese, and validated in other countries including Brazil. A recent study by da Cruz Ribeiro E Souza et al. showed that the Brazilian version of PsAID‐12 is a reliable and valid measure of the impact of the disease in patients with PsA and correlated with scores of disease activity assessment (da Cruz Ribeiro E Souza et al., 2020). These findings suggest that the PsAID may have widespread clinical utility in PsA populations worldwide.

## 4 EMERGING OUTCOME MEASUREMENTS

There is great interest in identifying biomarkers that may serve as surrogate endpoints, that is, as substitutes for clinically meaningful endpoints. To be considered a surrogate endpoint, there must be evidence that a biomarker consistently and accurately predicts a clinical outcome (Chandran and Scher., 2014). To date, it has been challenging to identify a single, or combination of biomarkers to aid treatment monitoring and outcome assessments in PsA patients. Alongside the inherent ‘user error’ associated with some clinical assessments, a biomarker would, in theory, offer a standardized and objective way of monitoring patients, and allow for potential easier head‐to‐head comparison of treatments.

To date, no single biomarker has been able to detect joint involvement in patients. However, at study in 2009 showed that the levels of the pro‐inflammatory cytokine IL‐6 correlated with joint involvement (Alenius et al., 2009). Studies have also shown that disease activity also correlates with CRP, IL‐16, calprotectin, IL‐12/IL‐23p40 and ICAM‐1 (Juneblad et al., 2018). Although studies have shown correlation between biomarker and disease activity, different PsA disease phenotypes are often associated with different biomarkers, suggesting that a panel of biomarkers may be a more suitable approach, rather than using a single readout.

Recently, this approach has been examined, with a study examining the screening of 107 proteins through a multiplex assay in order to identify potential biomarkers to predict responses to therapies (Ademowo et al., 2016). Fifty‐seven proteins were identified that correlated with disease, with the study providing evidence that a multiplexed protein assay of a panel of biomarkers that predict response to treatment could be developed.

Although still in its infancy, the early findings from these small studies suggest that a combinatorial approach of biomarkers may prove feasible in the near future for assessing disease activity and potentially response to therapies (Generali et al., 2016). The use of emerging technologies, such as smartphone applications like Psorcast, may help to further harness PROMs in a real‐world setting (Webster et al., 2020).

## 5 DISCUSSION

Psoriatic arthritis is a clinically heterogenous disease with a range of disease domains. Capturing disease across these domains and broad patient populations is challenging, and with new therapeutic options rapidly emerging, it is important that robust outcome measures are developed and validated to help assess the clinical meaningfulness of treatments. It is clear that at present, composite approaches, whether clinical or biomarker based, are likely the most meaningful in capturing disease activity and associated burden. Further alignment through working groups such as GRAPPA and OMERACT may help guide the validation and subsequent implementation of such outcome measurements.

## AUTHOR CONTRIBUTION

Simon Hackett wrote and reviewed the manuscript, Laura C. Coates also wrote and reviewed the manuscript.

## CONFLICTS OF INTEREST

None.

## ETHICS STATEMENT

This is a review so no ethical approval was needed.

## Data Availability

No data are contained within this review that have been generated by the authors.
